# Nonsense and missense mutation of mitochondrial ND6 gene promotes cell migration and invasion in human lung adenocarcinoma

**DOI:** 10.1186/s12885-015-1349-z

**Published:** 2015-05-02

**Authors:** Yang Yuan, Weixing Wang, Huizhong Li, Yongwei Yu, Jin Tao, Shengdong Huang, Zhiyong Zeng

**Affiliations:** 1 Department of Cardiothoracic Surgery, Changhai Hospital, Shanghai, P.R. China; 2 Department of Medical Imaging, Changhai Hospital, Shanghai, P.R. China; 3 Department of Pathology, Changhai Hospital, Second Military Medical University, Shanghai, P.R. China; 4 Department of Cardiothoracic Surgery, Fuzhou General Hospital of Nanjing Command, PLA, Nanjing, China

**Keywords:** Mitochondrial DNA, NADH dehydrogenase, Reactive oxygen species, Lung adenocarcinoma

## Abstract

**Background:**

Previous study showed that mitochondrial ND6 (mitND6) gene missense mutation resulted in NADH dehydrogenase deficiency and was associated with tumor metastasis in several mouse tumor cell lines. In the present study, we investigated the possible role of mitND6 gene nonsense and missense mutations in the metastasis of human lung adenocarcinoma.

**Methods:**

The presence of mitND6 gene mutations was screened by DNA sequencing of tumor tissues from 87 primary lung adenocarcinoma patients and the correlation of the mutations with the clinical features was analyzed. In addition, we constructed cytoplasmic hybrid cells with denucleared primary lung adenocarcinoma cell as the mitochondria donor and mitochondria depleted lung adenocarcinoma A549 cell as the nuclear donor. Using these cells, we studied the effects of mitND6 gene nonsense and missense mutations on cell migration and invasion through wounding healing and matrigel-coated transwell assay. The effects of mitND6 gene mutations on NADH dehydrogenase activity and ROS production were analyzed by spectrophotometry and flow cytometry.

**Results:**

mitND6 gene nonsense and missense mutations were detected in 11 of 87 lung adenocarcinoma specimens and was correlated with the clinical features including age, pathological grade, tumor stage, lymph node metastasis and survival rate. Moreover, A549 cell containing mitND6 gene nonsense and missense mutation exhibited significantly lower activity of NADH dehydrogenase, higher level of ROS, higher capacity of cell migration and invasion, and higher pAKT and pERK1/ERK2 expression level than cells with the wild type mitND6 gene. In addition, NADH dehydrogenase inhibitor rotenone was found to significantly promote the migration and invasion of A549 cells.

**Conclusions:**

Our data suggest that mitND6 gene nonsense and missense mutation might promote cell migration and invasion in lung adenocarcinoma, probably by NADH dehydrogenase deficiency induced over-production of ROS.

## Background

Lung cancer is one of the most common malignant tumors in the world [1,2]. According to the etiologic and pathologic characteristics, lung cancer could be divided into two main forms, small cell lung cancer (SCLC) and non-small cell lung cancer (NSCLC) [3]. The incidence of lung adenocarcinoma, a subtype of NSCLC, is by far the most prevalent lung cancer in China [2,4]. Although novel surgical treatment can prolong the survival time of the patients, the long-term survival rate of lung adenocarcinoma after surgery remains low [1,2]. Molecular prognostic factors of lung adenocarcinoma such as nuclear DNA mutations [5,6] have been investigated extensively in clinical samples. However, whether mitochondrial DNA (mtDNA) alteration is associated with tumor properties has not been explored vigorously.

Mammalian mitochondria are usually depicted as elongated cylindrical particles originated in ancestral eukaryotic cells through endosymbiosis of free living bacteria capable of metabolizing oxygen [7-9]. It is well known that the core functions of mitochondria include oxidative phosphorylation, amino acid metabolism, fatty acid oxidation, and ion homeostasis [7-9]. In recent years, mounting data suggest that mitochondria are involved in crucial cell properties such as proliferation, differentiation and apoptosis [10,11]. Most mammalian cells contain 10^3^ - 10^4^ copies of mtDNA and the mutation rate of mtDNA is much higher than that of nuclear DNA [7,8]. Mitochondrial dysfunction as a result of mtDNA mutation is increasingly recognized as an important cause of human disease [12]. MtDNA mutations have been identified in various types of tumors including lung adenocarcinoma [13].

MitND6 gene encodes ND6 subunit, which is one of the 40 subunits of the NADH dehydrogenase (also known as complex I), in mammalian cells [14]. In the past ten years, a variety of point mutations of ND6 gene were showed to affect NADH dehydrogenase activity [15-18] leading to NADH dehydrogenase deficiency, and were associated with maternally inherited diseases such as Leber’s hereditary optic neuropathy (LHON) [15,16] and mitochondrial encephalomyopathy with lactic acidosis and stroke-like episodes (MELAS) [17,18]. Using a cytoplasmic hybrid technology with several tumor cell lines, Ishikawa et al. [19] reported that ND6 missense mutation contribute to tumor cell metastasis in mouse fibrosarcoma, lung carcinoma and colon cancer. However, the biological role of mitND6 gene mutation in human lung adenocarcinoma cells has not been documented.

Here we set out to evaluate the role of mitND6 gene nonsense and missense mutation in human lung adenocarcinoma by clinical investigation and cellular experiments. Clinical investigation showed that mitND6 gene nonsense and missense mutation in lung adenocarcinoma cells was closely correlated with poor differentiation, advanced stage, lymph node metastasis of the tumor, and survival rate. With cytoplasmic hybrid cell (nuclear removed primary lung adenocarcinoma cell as mitochondria donor and mtDNA depleted A549 cell as nuclear donor), we further demonstrate that cells with mitND6 gene nonsense and missense mutation produced more reactive oxygen species (ROS) and exhibited higher capacities of migration and invasion. Our results suggest that mitochondrial ND6 gene nonsense and missense mutation in lung generation.

## Methods

### Lung adenocarcinoma specimens

A total of 87 primary lung adenocarcinoma patients that underwent pulmonectomy were enrolled in this study. Tumor specimens and paired normal lung tissue specimens taken from a site distant from the cancerous lesion were obtained from the consenting patients, as approved by the Medical Ethics Committee of Changhai Hospital and Fuzhou General Hospital and all patients gave written consent for use of tissue specimens. None of the patients received radiotherapy or chemotherapy before surgery. Clinical and pathological data including age, gender, pathological grading, tumor stage and lymph node metastasis were acquired from the medical records.

### Cell culture

Human lung adenocarcinoma cell line A549 was purchased from the Shanghai Institute of Biochemistry and Cell Biology (Shanghai, China). Cells were maintained in RPMI1640 (Invitrogen) supplemented with 10% fetal bovine serum (Invitrogen), 100 U/mL penicillin and 100 μg/mL streptomycin, within a humidified atmosphere containing 5% CO_2_ at 37°C.

Lung adenocarcinoma samples were mechanically dissociated and then digested in the medium containing 150 μg/mL Collagenase Type IV, 2 μg/mL DNase type I and 10 μg/mL hyaluronidase type V (Sigma) for 2 hr at 37°C. The resulting cell suspension was filtered through a 38-μm nylon mesh and single cells were harvested and subsequently seed in 25 cm^2^ flasks. At confluency, cells were subcultured following detachment by exposure to 0.25% trypsin for 2 min at 37°C. Cells were resuspended in fresh RPMI 1640 medium with 10% fetal bovine serum, within a humidified atmosphere containing 5% CO_2_ at 37°C.

### Sequencing of the Mitochondrial ND6 gene

The total cellular DNA of tissue samples were extracted using QIAamp genomic DNA kits (Qiagen). The DNA sample was kept at −20°C until use. The mitochondrial ND6 gene was PCR amplified (sence: ggcataattaaactttacttc; anti sence: catatcattggtcgtggttgtag) from the extracted DNA and then subjected to direct sequencing. The PCR conditions were set at 94°C for 2 minutes followed by 30 cycles of amplification at 94°C for 15 seconds, 58°C for 15 seconds, and 68°C for 40 seconds, and the final extension at 68°C for 6 minutes. PCR products were subject to electrophoresis on a 1.5% agarose gel to separate the DNA bands and visualized by ultraviolet light illumination after ethidium bromide staining. The DNA band of interest was then cut out of the gel and subject to direct sequencing. The results were compared in pairs with reference to the human mitochondrial genome database (http://www.mitomap.org/MITOMAP).

### Construction of cytoplasmic hybrid

Cytoplasmic hybrids were constructed as previously described [19]. Briefly, A549 cell subline without mitochondria (ρ0 A549 cells, nuclear donor) was constructed by 100 ng/mL ethidium bromide (EB) treating. Complete depletion of mtDNA was confirmed by PCR analysis. Cultured cells from primary lung adenocarcinoma (with and without ND6 gene mutations as mtDNA donor) were prepared by their pretreatment with cytochalasin B (10 μg/mL) at 37°C for 20 min and centrifugation at 9,000 × g at 37°C for 10 min. Resultant cytoplasts were fused with ρ0 cells by polyethylene glycol.

### NADH dehydrogenase activity analysis

Cells in log-phase growth were harvested, and the mitochondrial NADH dehydrogenase activity was detected with NADH Assay Kit (Abcam). Briefly, NADH and cytochrome c (oxidized form) were used as substrates for estimation of NADH dehydrogenase activity, and the reduction of cytochrome c was monitored at 550 nm.

### ROS production analysis

ROS generation was detected with mitochondrial superoxide indicator MitoSOX-RED (Invitrogen). Cells were incubated with 5 μM MitoSOX-RED for 10 min at 37°C in serum-free DMEM, washed twice with phosphate-buffered saline (PBS), and then immediately analyzed with a FACScan flow cytometer (Becton Dickinson, Mountain View, CA, USA). Those cells incubated with MitoSOX-RED and exhibited red fluorescence were determined as ROS positive cells.

### Wound healing assay

Lung adenocarcinoma cells were seeded on 6-well plates at a density of 5 × 10^5^ cells/well. After the cells reached sub-confluence, the mono-layer cells were wounded by scraping off the cells and then grown in medium for 72 hr. The migrated distance of cells was monitored and imaged under a microscope. The distances of cell migration were calculated by subtracting the distance between the lesion edges at 48 hr from the distance measured at 0 hr. The relative migrating distance of cells is measured by the distance of cell migration/the distance measured at 0 hr.

### Transwell assay

Cell migration and invasion were determined using a transwell (Costar) with a pore size of 0.8 μm. 5 × 10^3^ cells were seeded in serum-free medium in the upper chamber (normal chamber for migration assay and matrigel-coated chamber for invasion assay). The lower chamber was filled with medium containing 10% FBS. After incubating for 8 hr at 37°C, cells in the upper chamber were carefully removed with a cotton swab and the cells that had traversed to reverse face of the membrane were fixed in methanol, stained with Giemsa, and counted.

### Statistical analysis

Statistical significance was tested using SPSS15.0 software. For comparison of clinical features (except for aging) between patients with and without ND6 gene mutations, chi-square test was performed. The average age between the patients with and without mitND6 gene mutations was compared with Cochran & Cox t’ test. Other data are presented as mean ± SEM, using student t tests for 2-group comparison, and ANOVA for multi-groups comparison with Bonferroni’s post-test. A P value less than 0.05 is considered as statistically significant.

## Results

### MitND6 gene nonsense and missense mutations are correlated with age, pathological grade, tumor stage and lymph node metastasis in lung adenocarcinoma specimens

**Figure 1:**
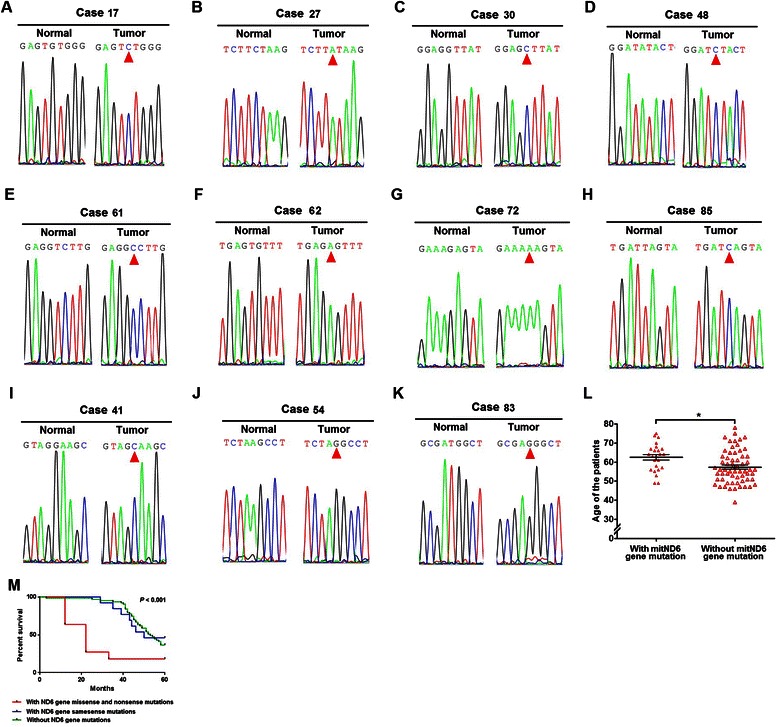
Mitochondrial ND6 gene nonsense and missense mutations in lung adenocarcinoma patients. **(A**-**K)** DNA sequencing electropherograms showing point mutations of mitochondrial ND6 gene lead to the amino acid changes of the protein in 11 cases. **(A**-**H)** represent missense mutation and **(I**-**J)** represent nonsense mutation. **(L)** The average age of the patients with or without mitND6 gene mutation. Each dot represents the age of a patient (n = 24 for patients with mitND6 gene mutation, n = 63 for patients without mitND6 gene mutation) with the line indicating the mean level; *, *P* < 0.05. **(M)** The survival rate of the patients with or without mitND6 gene mutation. n = 11 for patients with missense and nonsense mitND6 gene mutation, n = 13 for patients with samesense mitND6 gene mutation, n = 63 for patients without mitND6 gene mutation.

To determine the mitND6 gene mutations in lung adenocarcinoma specimens, we compared each of the mitND6 gene sequences of 87 patients to the Cambridge Reference Sequence. A total of 26 mitND6 gene mutations existed in 24 lung adenocarcinoma specimens (22 samples with one mitND6 gene mutation and 2 samples with two mitND6 gene mutations). As shown in Figure 1A-K, 8 mitND6 gene mutations were missense mutations that results in amino acid change of ND6 protein, 3 were nonsense mutations that results in premature termination of the translation of ND6 protein, and the other mutations were samesense mutations that render the ND6 protein sequence unaltered. In addition, the average age of the patients with mitND6 gene mutations was significantly higher than those without mitND6 gene mutations (Figure 1L). As shown in Table 1, mutations were more common in patients over 60 years of age, but there was no significant gender difference in incidence of mitND6 mutations. We further analyzed the relationship of mitND6 gene mutation with the clinical features including pathological grade, tumor stage and lymph node metastasis. It was found that missense and nonsense, but not samesense, mitND6 gene mutations were significantly correlated with the pathological grade, tumor stage and lymph node metastasis (Table 1). Moreover, ND6 gene missense and nonsense mutations were associated with shorter survival rate, whereas survival rate of patients with ND6 gene samesense mutations was not significantly different from those without ND6 gene mutations (Figure 1M). These results suggest that the mitND6 gene nonsense and missense mutation might be involved in the regulation of metastasis of lung adenocarcinoma.

**Table 1:** **Relationship between mitochondrial ND6 gene mutation and clinical features of lung adenocarcinoma**

Clinical features	Lung adenocarcinoma samples				
	With ND6 gene missense and nonsense mutations	With ND6 gene samesense mutations	Without ND6 gene mutations	Test	*P*
**Total**	11	13	63		
**Age (year)**					
<60	3/11	5/13	41/63	7.423	0.024
≥60	8/11	8/13	22/63		
**Gender**					
Male	7/11	9/13	43/63	0.106	0.949
Female	4/11	4/13	20/63		
**Pathological grading**					
Well	1/11	4/13	25/63	11.993	0.017
Moderately	4/11	7/13	30/63		
Poorly	6/11c	2/13	8/63		
**Tumor stage**					
T1/T2	2/11	8/13	37/63	6.547	0.038
T3/T4	9/11	5/13	26/63		
**Lymph node metastasis**					
Negative	2/11	9/13	38/63	7.794	0.020
Positive	9/11	4/13	25/63		

### MitND6 gene nonsense and missense mutations promote migration and invasion of lung adenocarcinoma cell line

To study the effect of mitND6 gene nonsense and missense mutation on cell migration and invasion, we constructed A549 sublines expressing mitND6 gene with missense and nonsense mutations by cytoplasmic hybrid technology [19]. Hybrid A549 cells containing normal mitochondrial, as well as normal A549 cells treated with rotenone, were used as controls.

**Figure 2:**
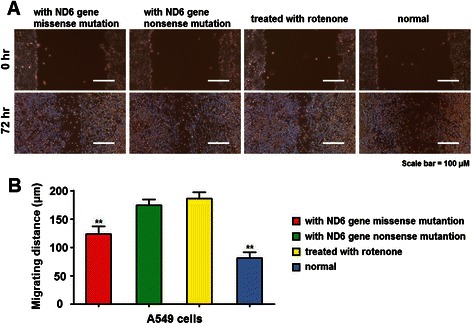
Mitochondrial ND6 gene nonsense and missense mutation promotes the migration of lung adenocarcinoma cells. **(A)** Representative images of different groups of A549 cells taken immediately (0 hr) and 72 hr after lesion in the wound healing assay. **(B)** Histogram showing the relative migration distance of cells. Bars = 100 μm. Data represent mean ± SEM from 4 independent experiments; **, *P* < 0.01.

Through wound healing assay, we found that the migrating distance of cells was significantly longer in cells with mitND6 gene nonsense and missense mutations and in cells treated with rotenone as compared to those with the wild type mitND6 gene (Figure 2A). Further, the mean migrating distance of cells with mitND6 gene nonsense mutation and cells treated with rotenone was similar and was significantly longer than cells with mitND6 gene missense mutation (Figure 2B).

**Figure 3:**
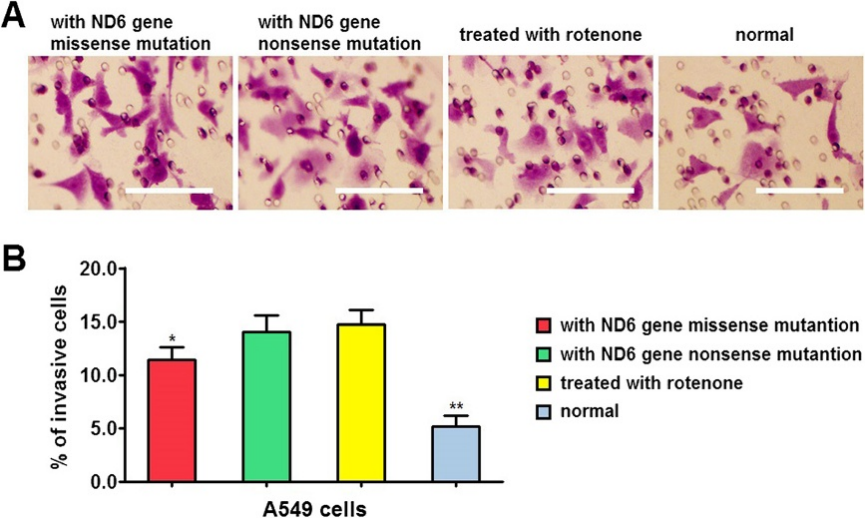
Mitochondrial ND6 gene nonsense and missense mutation promotes the invasion of lung adenocarcinoma cells. **(A)** Representative photographs showing invasion of different groups of A549 cells in the transwell assay. The invaded cells were stained with crystal violet. **(B)** Histogram showed the percentage of invasive cells. Bars = 50 μm. Data represent mean ± SEM from 4 independent experiments; **, *P* < 0.01.

In matrigel-coated transwell assay, the percentages of cells invaded through the matrigel was significantly higher in cells with mitND6 gene missense mutation than cells with normal mitND6 gene, and was further higher in cells with mitND6 gene nonsense mutation and in cells treated with rotenone. Cell invasion was not significantly different between mitND6 nonsense mutation group and rotenone-treated group (Figure 3).

These results suggest that nonsense and missense mutation of mitND6 gene, like inhibitor of NADH dehydrogenase, might promote migration and invasion of lung adenocarcinoma cells.

### MitND6 gene nonsense and missense mutation inhibit the activity of NADH dehydrogenase, increase production of ROS, and increases the activity of AKT and ERK/MAPK in lung adenocarcinoma cell line

**Figure 4:**
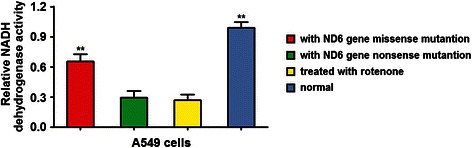
MitND6 gene nonsense and missense mutations inhibit the activity of NADH dehydrogenase. NADH dehydrogenase were analyzed by spectrophotometry. The histogram illustrates reduced NADH dehydrogenase activity in cells with nonsense and missense mutations of mitND6 gene and cells treated with rotenone compared to A549 cells with the wild type mND6 (normal). Data represent mean ± SEM from 4 independent experiments; **, *P* < 0.01.

Since ND6 is a subunit of the NADH dehydrogenase, we detected the effect of mitND6 gene nonsense and missense mutation on the activity of NADH dehydrogenase in lung adenocarcinoma cells. As shown in Figure 4, NADH dehydrogenase activity was reduced in A549 cells with mitND6 gene missense mutation (p < 0.05 vs. cells with normal mitND6 gene), and further reduced in cells with mitND6 gene nonsense mutation and in cells treated with rotenone (p < 0.01 and p < 0.05, respectively, vs. cells with normal mitochondrial ND6 gene). In addition, NADH dehydrogenase activity was not significantly different between mitND6 nonsense mutation and rotenone-treated cell groups. Hence, mitND6 gene nonsense and missense mutation resulted in reduced NADH dehydrogenase activity in lung adenocarcinoma cells.

**Figure 5:**
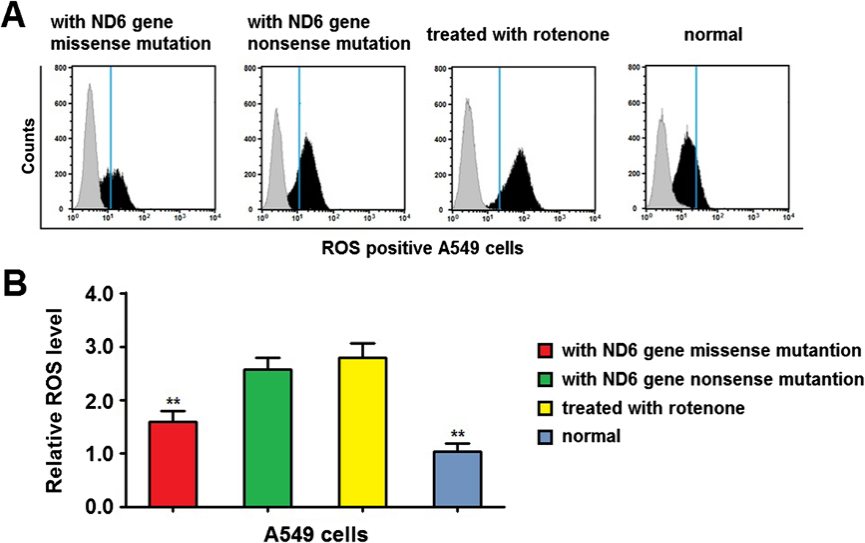
Mitochondrial ND6 gene nonsense and missense mutation over-produces the ROS. ROS level was analyzed by flow cytometry. **(A)** Representative flow cytometry graphs of A549 cells with the wild type mND6 (normal), cells with missense mutation and nonsense mutations of mitND6 gene and cells treated with rotenone. Histogram represented the ROS level in cells. **(B)** Histogram showed the relative ROS level of cells. Data represent mean ± SEM from 4 independent experiments; **, *P* < 0.01.

To study the role of mitochondrial ND6 gene nonsense and missense mutation in the regulation of cell migration and invasion, we further investigated the cellular ROS level, since it was reported that defective activity of NADH dehydrogenase could induce the increase of ROS [7-9] and the level of ROS is relevant to tumor cell migration, invasion and metastasis [11,19]. As shown in Figure 5A, the cellular ROS level was significantly higher in A549 cells with mitochondrial ND6 gene nonsense and missense mutations and cells treated with rotenone as compared to those with normal mitochondrial ND6 gene. Further, cells with mitochondrial ND6 gene nonsense mutation and cells treated with rotenone showed significantly higher cellular ROS level than cells with mitochondrial ND6 gene missense mutation, while there was no significantly difference between the last 2 groups (Figure 5B). These results suggest that the mitochondrial ND6 gene nonsense and missense mutation increase production of ROS in lung adenocarcinoma cells.

**Figure 6:**
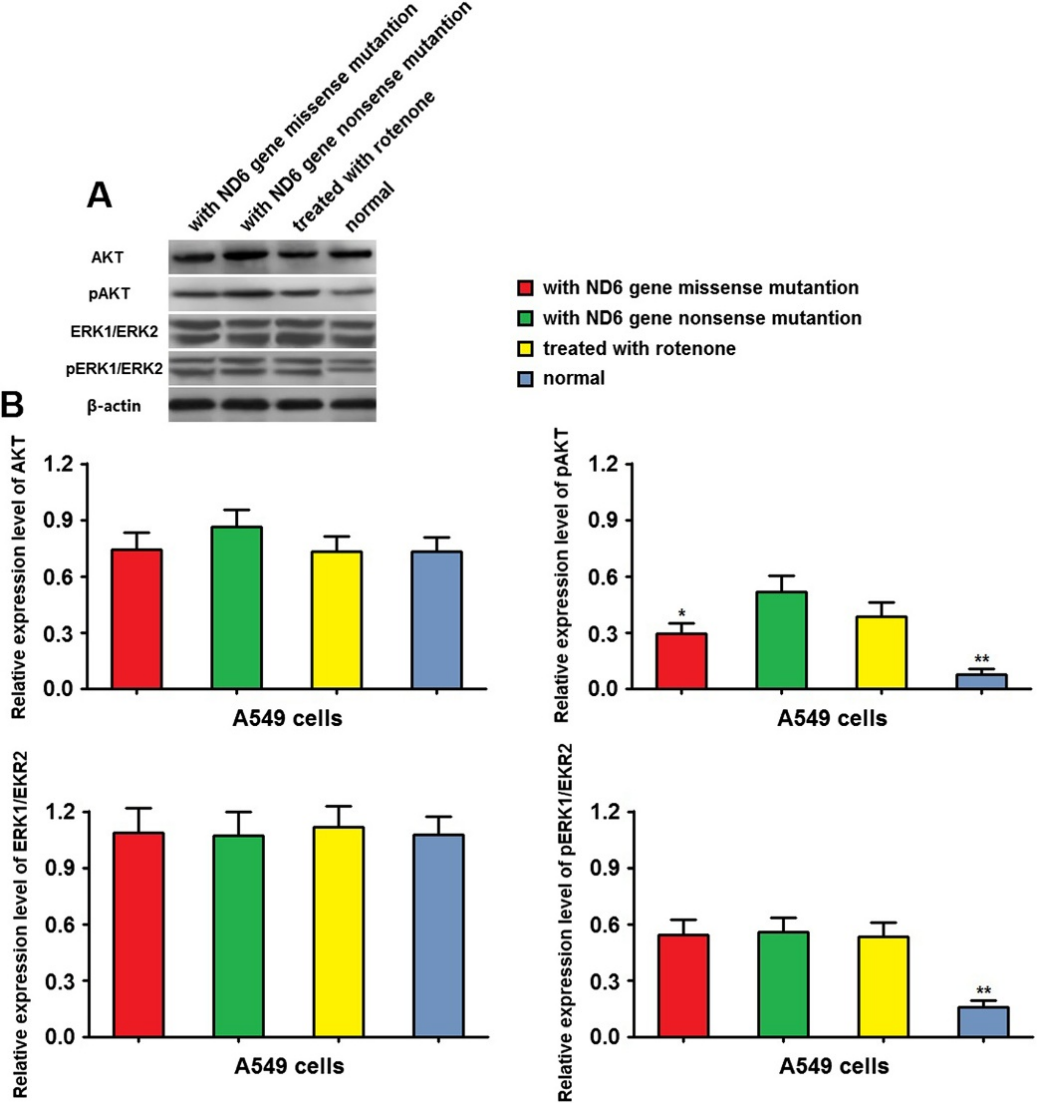
Mitochondrial ND6 gene nonsense and missense mutation increases the activity of AKT and ERK/MAPK. AKT and ERK1/ERK2 expression level was determined by western blot. **(A)** Representative graphs of total and phosphate AKT and ERK1/ERK2 expression of A549 cells with the wild type mND6 (normal), cells with missense mutation and nonsense mutations of mitND6 gene and cells treated with rotenone. **(B)** Histogram showed the relative total and phosphate AKT and ERK1/ERK2 expression level of cells Data represent mean ± SEM from 4 independent experiments; *, *P* < 0.05; **, *P* < 0.01.

ROS was reported to regulate tumor cell metastasis through AKT and ERK/MAPK signal pathway [20,21]. In the present study, our western blot results showed that cells containing mitND6 gene nonsense and missense mutation increased the expression level of phosphate AKT and ERK1/ERK2 (Figure 6A and B), but had no effect on total AKT and ERK1/ERK2 (Figure 6A and B). These results suggest that the mitochondrial ND6 gene nonsense and missense mutation increases the activity of AKT and ERK/MAPK in lung adenocarcinoma cell line.

## Discussion

The major finding of the present study is that nonsense and missense mutations of mitND6 gene are correlated with the clinical features of lung adenocarcinoma and that in adenocarcinoma cell lines (A549 cells) such mutations lead to reduced NADH dehydrogenase activity, increased ROS production and concomitantly promote cell migration and invasion.

As the only extranuclear source of DNA in animal cells, mitochondrial genome has unique characteristics different from nuclear genomic DNA [7-9]. MtDNA is not protected by histones, lacks nucleotide excision repair is located in an environment with high levels of potentially damaging ROS [22,23], and hence may be more prone to mutations than nuclear DNA. Consistently, it was reported that the rate of human mtDNA mutation was approximately 17 times greater than that of nuclear DNA [24]. mtDNA mutations have been detected in different types of tumors including lung adenocarcinoma. Ishigawa et al. have shown that mtDNA missense mutations might promote cell invasion and metastasis in several mice tumor cell lines [19]. However, there still is a lack of investigation into the relationship between mitochondrial gene mutations and the clinical features of tumors.

 In the present study, point mutations of mitND6 genes were detected in 27.5% lung adenocarcinoma samples with 45.8% of the mutations being nonsense and missense mutations that result in amino acid changes of the protein. Importantly, nonsense and missense mutations of mitND6 were associated with poor differentiation grading, advanced tumor stage, lymph node metastasis and shorter survival rate, suggesting that mitND6 gene mutation may play an important role in regulating the progression and metastasis of lung adenocarcinomas. However, whether the status of mitND6 mutations is an independent factor predicting metastasis in lung cancer warrants further analysis with more patients’ samples. In addition, we found that mitND6 gene mutations were more common in lung adenocarcinoma patients over 60 years of age, and the average age was significantly higher in the patients with mitND6 gene mutations as compared to those without mitND6 gene mutations. It was reported that cells accumulate somatic mutations of mitochondrial DNA as part of normal ageing. These data are consistent with the previous reports that mtDNA mutations are accumulated during aging process [25,26].

Tumor metastasis is the major cause of low survival rate of lung adenocarcinoma patients [27,28]. Previous studies showed that the cybrid cells containing the missense mutant mitND6 gene derived from the highly metastatic mouse tumor cell lines conferred higher metastatic potential, regardless of the nuclear background [19]. Consistently, here we demonstrated that mitND6 gene nonsense and missense mutation significantly promote the migration and invasion of human lung adenocarcinoma cell line A549. In addition, cells containing mitND6 nonsense mutation showed significantly higher migrate and invasive capacity than cells containing mitDN6 missense mutation. On the other hand, our clinical data indicated that the mitND6 gene nonsense and missense mutation was significantly correlated with lymph node metastasis of lung adenocarcinoma. These results suggest that mitochondrial ND6 gene nonsense and missense mutation may contribute to the metastasis of lung adenocarcinoma.

ROS could function as signaling molecule to stimulate tumor cell proliferation, promote genetic instability, and contribute to carcinogenesis by affecting the expression and activity of certain redox sensitive molecules. It is well known that mitochondria is the major site of ROS generation occurring mainly at NADH dehydrogenase of the respiratory chain [7,9]. Recently, Ishikawa et al. reported that mitND6 gene missense mutation induced NADH dehydrogenase defects and in turn increased ROS production in some mice tumor cell lines [19]. However, the effect of mitND6 gene nonsense and missense mutation on the ROS production in human lung adenocarcinoma cells has not been identified before. In the present study, we found that mitND6 gene nonsense and missense mutation resulted in inhibition of NADH dehydrogenase activity and significantly higher levels of ROS in A549 cells. In addition, cells containing mitND6 nonsense mutation exhibited significantly lower activity of NADH dehydrogenase and significantly higher level of ROS than cells containing mitDN6 missense mutation, and showed no significant differences to those treated with rotenone (an inhibitor of NADH dehydrogenase). It was reported that treated cancer cells with diphenyliodonium, a specific inhibitor of ROS generation, could reverse the promotion of migration and invasion by ROS, suggesting that ROS plays role in the regulation of cell migration and invasion in cancer cells [20]. Combined with the results that mitND6 gene nonsense and missense mutation could inhibit of NADH dehydrogenase activity and increase the level of ROS in A549 cells, we suggest that mitochondrial ND6 gene nonsense and missense mutations may induce ROS over-production by reducing the activity of NADH dehydrogenase and further regulate cell migration and invasion in lung adenocarcinoma cells.

It was reported that increased generation of ROS could promote the cell invasion and tumor metastasis in some types of malignances including lung adenocarcinoma [29]. Consistent with the previous findings in human breast cancer [30], we found that rotenone caused dramatic increase in ROS production and concomitant increases in migration and invasion of A549 cells, suggesting that dysfunction of NADH dehydrogenase may enhance the metastatic potential of lung adenocarcinoma cells. Importantly, mitND6 gene nonsense and missense mutation resulted in inhibition of NADH dehydrogenase activity, significantly higher levels of ROS, and increasing the capacities of migrating and invasion in A549 cells. ROS have been shown to play a role in the AKT and ERK/MAPK activation, which plays essential roles in tumor cells, and activation of these kinases may increase the capacity of tumor cell migration, invasion and metastasis [20,21]. Our results show that mitND6 gene nonsense and missense mutation could increase the expression level of phosphate AKT and ERK1/ERK2, but had no effect on total AKT and ERK1/ERK2. Based on these findings, we propose that mitochondrial ND6 gene nonsense and missense mutation may contribute to the metastasis of lung adenocarcinoma by reducing the activity of NADH dehydrogenase and in turn the over-production of ROS probably through AKT and ERK1/ERK2 signal pathway.

## Conclusions

In summary, we show here high incidence of nonsense and missense point mutations of mitND6 gene in lung adenocarcinoma, especially in patients over 60 years of age. Such mutations were correlated with the pathological grade, stage and lymph node metastasis of tumors. In lung adenocarcinoma cell line (A549), nonsense and missense mitND6 gene mutations resulted in significant decrease in NADH dehydrogenase activity, increases in ROS production and promoted cell migration and invasion. These findings suggest that mitND6 gene nonsense and missense mutations may contribute to the metastasis of human lung adenocarcinoma.
